# Online Partner Seeking and Sexual Behaviors Among Men Who Have Sex With Men From Small and Midsized Towns: Cross-sectional Study

**DOI:** 10.2196/35056

**Published:** 2022-06-10

**Authors:** Vira Pravosud, April M Ballard, Ian W Holloway, April M Young

**Affiliations:** 1 Center for Tobacco Control Research and Education Cardiovascular Research Institute University of California, San Francisco San Francisco, CA United States; 2 Gangarosa Department of Environmental Health Emory University Rollins School of Public Health Atlanta, GA United States; 3 Luskin School of Public Affairs University of California, Los Angeles Los Angeles, CA United States; 4 Department of Epidemiology College of Public Health University of Kentucky Lexington, KY United States; 5 Center on Drug and Alcohol Research Department of Behavioral Science University of Kentucky College of Medicine Lexington, KY United States

**Keywords:** men who have sex with men, MSM, sexual risk behaviors, social networking and dating apps, online tools, HIV, sexually transmitted infection, STI prevention, mobile phone

## Abstract

**Background:**

Men who have sex with men (MSM) residing outside of large urban areas are underrepresented in research on online partner seeking and sexual behaviors related to transmission of HIV.

**Objective:**

We aimed to determine associations between the use of the internet or social networking apps (*online tools*) to meet partners for sex, dating, or for both purposes (*online partner seeking*) and sexual behaviors among MSM residing in small and midsized towns in Kentucky, United States.

**Methods:**

Using peer-referral sampling and online self-administered questionnaires, data were collected from 252 men, aged 18 to 34 years, who had recently (past 6 months) engaged in anal sex with another man and resided in Central Kentucky. Using multivariable logistic regression models, we assessed associations of online partner seeking and HIV-related sexual behaviors.

**Results:**

Most (181/252, 71.8%) of the participants reported using online tools for partner seeking. Of these 181 respondents, 166 (91.7%) had used online tools to meet partners for sex (n=45, 27.1% for sex only; and n=121, 72.9% for sex and dating) and 136 (75.1%) had used online tools to meet partners for dating (n=15, 11% for dating only; and n=121, 89% for sex and dating). Adjusted analyses revealed that MSM who had engaged in condomless insertive and receptive anal intercourse were less likely to report online partner seeking (adjusted odds ratio [aOR] 0.22, 95% CI 0.07-0.68; *P*=.009 and aOR 0.25, 95% CI 0.10-0.66; *P*=.005, respectively). Increased number of insertive and receptive anal sex partners and substance use before or during sex were associated with higher odds of online partner seeking (aOR 1.31, 95% CI 1.11-1.55; *P*=.001; aOR 1.20, 95% CI 1.05-1.39; *P*=.008; and aOR 2.50, 95% CI 1.41-4.44; *P*=.002, respectively).

**Conclusions:**

Among MSM who reside outside of large urban areas and practice online partner seeking, HIV risk-reduction interventions should address safer sex practices, including the risks for HIV transmission associated with alcohol or drug use before or during sex. MSM who do not practice online partner seeking are in need of continued outreach to reduce condomless anal sex.

## Introduction

### Background

Men who have sex with men (MSM) have the highest burden of HIV in the United States [1], with almost 70% of the new HIV cases being attributable to male-to-male sexual contact [1]. Although prior research has contributed immensely to our understanding of prevention of HIV transmission among MSM, new avenues for partner seeking among MSM have emerged over the past 3 decades, including through the internet, social media, and geosocial networking apps.

The use of the internet and social media can facilitate health-protective and safer sex practices among MSM who seek partners online [2,3]. Internet profiles become a place for MSM to disclose attitudes toward substance use [4] and HIV status [2,4-7], as well as inform others whether they take pre-exposure prophylaxis (PrEP) for HIV prevention [8]. Such practices may help internet-using MSM to make more informed sexual health decisions [2,5,7]. As described in previous research some internet-using MSM are more likely to report HIV testing [9] and condom use during anal intercourse with partners met online [10-12].

However, online partner seeking has also been associated with increased engagement in HIV-related risk behaviors [9-11,13-20], including an increased number of sex partners [11,17,18,21], higher prevalence of substance use [9,22], condomless anal intercourse with male partners [13,18,19], and casual sex with HIV-positive partners met online [16]. Compared with meeting partners in person, online partner seeking among MSM has also been associated with potential transmission of HIV [23] and greater odds of testing positive for sexually transmitted infections (STIs; eg, syphilis [24], gonorrhea, and chlamydia [22]), but the factors driving these associations are not well understood.

Most studies to date have described associations between the use of online partner seeking and sexual risk behaviors among MSM residing in large metropolitan centers; for example, Los Angeles [11,22,25], New York City [7,18,26,27], and San Francisco [28] in the United States. Few studies (including those published more than 10 years ago [29-31]) have examined patterns of internet use and associated sexual risk behaviors among MSM from rural areas [29-32] or small and midsized towns [33]. Similar to findings from large metropolitan areas [22], rural MSM who used the internet to search for sex partners had a higher prevalence of condomless anal intercourse [30,31]. Evidence shows that MSM from rural communities can be especially susceptible to social estrangement [34] and hostility [35], as well as sexual isolation and stigma [36,37], and that rural MSM often have few identifiable venues where they can meet other MSM [33,35,38,39]. MSM who reside outside of large urban areas may use social networking and dating websites or mobile apps as a safe and convenient way to meet partners [35] and travel from, or to, nearby metropolitan centers to meet people contacted online [35,40,41]. These findings highlight the need for additional research on sexual behaviors and the use of various online tools for partner seeking among MSM across the urban and nonurban continuum.

### The Goal of This Study

As noted [42], MSM from small and midsized towns have been underrepresented in research, leaving a gap in knowledge that is concerning especially among those who reside in the American South, a region that remains disproportionately affected by HIV [1,43]. As there is a need for more geographic diversity in studies on the topic, we focused on MSM residing in 15 Central Kentucky counties that predominantly consist of small and midsized towns [44,45] and are located in the Bluegrass Area Development District [46]. In 2019, the Bluegrass Area Development District had the second greatest percentage of HIV diagnoses (19%) in the state of Kentucky [47], where the majority of cases among men were diagnosed in MSM (67%), persons who inject drugs (PWID; 8%), or MSM and PWID (6%) [47]. The relevance of the region to HIV research also lies in the region’s geographic proximity to rural Central Appalachia, which is considered highly vulnerable to an HIV outbreak associated with injection drug use [48], and to 2 recent HIV clusters in Northern Kentucky [49,50] and West Virginia [51], wherein most cases were among PWID [52] and PWID with male-to-male sexual contact [53]. In 2020, Kentucky was named 1 of just 7 rural states targeted by the Ending the HIV Epidemic initiative based on the percentage of new diagnoses occurring in rural areas [54]. Therefore, the aim of this exploratory study was to describe online partner seeking among MSM residing in small and midsized towns in Kentucky and to compare HIV sexual risk behaviors in this understudied population between those who use online tools for partner seeking and those who do not.

## Methods

### Study Design and Data Collection

From February 2018 to July 2018, a total of 253 participants were recruited using targeted street outreach and web-based respondent-driven sampling, which was previously shown to be an effective technique for reaching stigmatized populations, including MSM [55]. The recruitment process for the study is described in more detail elsewhere [56]. Briefly, the research team posted flyers on social media through a study-specific page and young adult lesbian, gay, bisexual, transgender, and queer groups and distributed flyers at various local venues that were outwardly friendly to members of the lesbian, gay, bisexual, transgender, and queer community (including adult entertainment stores, bars, and health clinics) as well as at 2 local pride festivals. All flyers directed individuals to the study website, where a link to the study’s online Qualtrics survey was posted. Upon completion of the survey, participants were offered a US $25 incentive in the form of an e-gift card or mailed payment [56]. Participants could refer up to 3 peers and receive up to US $30 if their referrals were eligible and completed the survey [56].

On the basis of demographic and behavioral factors associated with new HIV infections, the eligibility criteria included being male, aged 18 to 34 years, having engaged in anal sex with another man during the past 6 months, and residing in 1 of 15 Central Kentucky counties. The study specifically focused on young MSM because they experience the highest burden of HIV [57]. In total, 787 screening questionnaires were received, among which 490 (62.2%) were eligible and 414 (52.6%) were complete survey entries. Recent studies have demonstrated that online research may be at risk of fraudulent or invalid responses [58,59]. As such, the study staff implemented a rigorous fraud prevention and detection mechanism to ensure data quality based on check of geolocation, telephone number, name, email address, personal information, and survey duration [56]. The protocol also included direct outreach by staff to those deemed potentially fraudulent. Of the 414 responses considered, after implementing our fraud-detection strategy, we excluded 161 (38.9%) responses, leaving 253 (61.1%) valid responses [56]. The 253 participants screened eligible by checking that they had engaged in anal sex with another man in the past 6 months. However, during further data cleaning, we identified that, of these 253 participants, 1 (0.4%) contradicted their screening answer in their response of a zero value for the question about the number of lifetime male partners. Thus, this participant was excluded. The final analytic sample included 252 participants who were recruited online, through flyers, or through in-person outreach activities.

### Ethics Approval

The institutional review board at the University of Kentucky approved the study protocol (76147). Survey participants completed informed consent forms [56].

### Measures

#### Outcome Variable

As many social networking and dating sites have both webpages and smartphone-based apps, online partner seeking in this study is defined as the use of either type of online platform for dating or sex. The outcome was defined as an affirmative response to at least one of the following 2 survey questions: “In the past 6 months, have you used any social media tools or apps to meet people for sex?” and “In the past 6 months, have you used any social media tools or apps to meet partners to date, not necessarily just for sex?” Given their significant correlation (*χ*^2^_1_=70.1; *P*<.001), the answers were combined in an aggregate outcome: online partner seeking, which was a binary variable (yes or no).

#### Demographic Characteristics

Participants provided information on various demographic characteristics, including age, gender, race, ethnicity, sexual orientation, highest level of education completed, income in the past 30 days (in US $), and county of residence. Because of the sample’s limited heterogeneity on gender, race and ethnicity, sexual orientation, and education, these variables were recoded into 2-level categories representing, respectively, man versus other gender groups; White and non-Hispanic versus other racial and ethnic groups; gay or mostly gay versus other sexual orientation; high school or General Educational Development Test (GED) or less versus (some) college degree or higher. Rural-Urban Continuum Codes [44] were used to classify counties of residence as metropolitan (5 counties, codes 1-3) and nonmetropolitan (6 counties, codes 4-6). Of note, the metropolitan counties were far smaller than the metropolises that have been the focus of previous research (eg, Los Angeles and San Francisco); the largest county had a population of less than 325,000 [45] and encompassed a midsized town. The other 4 metropolitan counties ranged in population size from 24,939 to 48,586 [44].

#### Behavioral Characteristics and STI and HIV Status

The survey included questions about lifetime behaviors such as sex with HIV-positive partners, HIV testing, antiretroviral therapy, and PrEP use among the respondents. The survey also asked about recent (past 6 months) drug use and engagement in sexual behaviors, including types of intercourse (insertive only, receptive only, and either insertive or receptive), number of male anal sex partners with whom the participant was the insertive (ie, was top) or receptive (ie, was bottom) partner, and frequency of condom use in receptive and insertive positions. Condomless insertive and receptive anal intercourse were analyzed as 2 binary variables created based on responses to questions that asked participants to report the percentage of recent anal sex acts in which a condom was used. Because of kurtosis in the distribution for continuous variables as well as the HIV risk entailed in any condomless sex [60], the variables were dichotomized where condomless insertive or receptive anal intercourse was defined as <100% condom use during insertive or receptive sex acts in the past 6 months (similar to previous research [61,62]). Of note, the survey did not ask participants to specify the HIV status of their partners. In addition, we examined recent engagement in group sex and use of alcohol or any drug (including marijuana, prescription drugs to get high, or any other illicit drug) before or during sex. The survey also asked whether participants had ever been diagnosed with STIs (chlamydia, gonorrhea, oral or anal herpes, or syphilis) or tested positive for HIV in their lifetime.

### Statistical Analysis

SAS software (version 9.4; SAS Institute Inc) was used to conduct all analyses. Unadjusted odds ratios (ORs) and adjusted ORs (aORs) and 95% CIs obtained from bivariate and multivariable logistic regression models, respectively, were used as the measures of association between online partner seeking and demographic and behavioral characteristics. We estimated 3 models examining the association between online partner seeking and 3 behaviors relevant to HIV transmission based on previous literature [60,63-66] that were significantly (*P*<.05) associated with online partner seeking in bivariate analyses and had a sufficient cell size for analysis: condomless insertive anal intercourse (model 1) and condomless receptive anal intercourse (model 2) with male partners, as well as substance use before or during sex (model 3).

On the basis of findings from previous research about differences in online partner seeking by demographics as well as results of diagnostics of confounding by demographic variables in our analysis, final models were adjusted for age [9,67], education [9], and race and ethnicity [9,15,25,68,69]. Models focused on estimating the association between online partner seeking and condomless insertive or receptive anal intercourse were also adjusted for the number of male insertive or receptive partners in the past 6 months and PrEP use in the lifetime. We examined all possible 2-way interactions for each model with their corresponding covariates and found no significant interactions. In addition, we tested for multicollinearity in all adjusted models and identified no multicollinearity. We also conducted sensitivity analyses while adjusting all models for residence in metropolitan and nonmetropolitan counties.

We used complete case analysis for each of the final multivariable regression models. Examination of missing versus included observations revealed no associations between missingness and most of the covariates of interest and no differences regarding the values of the dependent variable, online partner seeking.

## Results

### Characteristics of Survey Participants

Of the 252 respondents, 205 (81.3%) were White and non-Hispanic, 244 (96.8%) identified as male, and 215 (85.3%) self-identified as gay or mostly gay. The median age of participants was 25.8 (IQR 22.8-29.2) years. Of the 252 respondents, 216 (85.7%) were in college or had obtained an undergraduate degree or higher; in addition, 214 (84.9%) were currently residing in a metropolitan county, whereas 38 (15.1%) resided in nonmetropolitan areas (Table 1).

Of the 181 respondents who reported using online tools for partner seeking, 166 (91.7%) had used online tools to meet partners for sex (n=45, 27.1% for sex only; and n=121, 72.9% for sex and dating) and 136 (75.1%) had used online tools to meet partners for dating (n=15, 11% for dating only; and n=121, 89% for sex and dating). Overall, 71.8% (181/252) of the respondents had used online tools to meet other men for sex or dating: 24.9% (45/181) used online tools to meet people for sex only, 8.3% (15/181) used online tools to meet people exclusively for dating, and 66.9% (121/181) used online tools for both sex and dating. More than 20 online tools were listed by the 181 MSM who practiced online partner seeking, including Grindr (n=154, 85.1%), Tinder (n=93, 51.4%), Facebook (n=57, 31.5%), Scruff (n=53, 29.3%), and Adam4Adam (n=28, 15.5%), as well as others such as Backpage, Bumble, Craigslist, eHarmony, GROWLr, Hornet, Hot or Not, Instagram, Jack’d, Manhunt, Match, Meetme, OK Cupid, Plenty of Fish, Snapchat, Tumblr, Twitter, and VGL.

**Table 1:** Demographic, health, and behavioral characteristics of the respondents (N=252).

Characteristics or behavior				Values^a^
**Demographic characteristics**				
	Age (years), median (IQR)			25.8 (22.8-29.2)
	Income in the past 30 days (US $), median (IQR)			1600 (800-2500)
	**Gender, n (%)**			
		Man	244 (96.8)	
		Other (transgender man or woman; gender queer)	8 (3.2)	
	**Race, n (%)**			
		White	211 (83.7)	
		Black or African American	20 (7.9)	
		Other (Pacific Islander, biracial, or multiracial)	18 (7.1)	
	**Ethnicity, n (%)**			
		Non-Hispanic	235 (93.3)	
		Hispanic	12 (4.8)	
	**Race and ethnicity, n (%)**			
		White and non-Hispanic	205 (81.3)	
		Other	40 (15.9)	
	**Sexual orientation, n (%)**			
		Gay or mostly gay	215 (85.3)	
		Other (bisexual or heterosexual or pansexual or queer)	36 (14.3)	
	**Education, n (%)**			
		Less than or some high school or high school graduate or GED^b^	36 (14.3)	
		Some college or college graduate or higher	216 (85.7)	
	**County of residence^c^, n (%)**			
		Metropolitan	214 (84.9)	
		Nonmetropolitan	38 (15.1)	
**Recent behaviors (past 6 months)**				
	**Type of anal sex with male partners, n (%)**			
		Only insertive (ie, participant was always in insertive position)	59 (23.4)	
		Only receptive (ie, participant was always in receptive position)	44 (17.5)	
		Insertive or receptive	148 (58.7)	
	Number of male insertive anal sex partners^d^, median (IQR)			1 (1-3)
	Number of male receptive anal sex partners^e^, median (IQR)			1 (1-4)
	Number of male oral sex partners, median (IQR)			3 (1-8)
	Condomless insertive anal intercourse^d^, n (%)			180 (71.4)
	Condomless receptive anal intercourse^e^, n (%)			170 (67.5)
	Engagement in group sex, n (%)			63 (25)
**Recent substance use (past 6 months), n (%)**				
	Used drugs to get high			144 (57.1)
	Daily use of drugs to get high			33 (13.1)
	Alcohol or any illicit drug use before or during sex			151 (59.9)
	Illicit drug use before or during sex with people met through social media (N=181; not available: 71)			65 (35.9)
**Lifetime behaviors, n (%)**				
	Ever had sex with HIV-positive partners			54 (21.4)
	Ever used drugs to get high in the lifetime			192 (76.2)
	Ever injected drugs in the lifetime			14 (5.6)
**HIV or STI^f^ status and PrEP^g^ use, n (%)**				
	Ever tested positive for STIs			72 (28.6)
	Ever been tested for HIV			215 (85.3)
	Ever tested positive for HIV			17 (6.7)
	Antiretroviral therapy uptake			17 (6.7)
	Ever used PrEP			37 (14.7)^h^

^a^Percentages are out of the total sample and may not add to total 100% because of rounding to 1 decimal. Sample sizes for some variables could vary because of missing data as well as refuse to answer or not applicable (n/a) responses.

^b^GED: General Educational Development Test.

^c^The US Department of Agriculture 2013 Rural-Urban Continuum Codes were used to classify counties of residence as metropolitan (5 counties, codes 1-3) and nonmetropolitan (6 counties, codes 4-6).

^d^With whom participants were in the insertive position.

^e^With whom participants were in the receptive position.

^f^STI: sexually transmitted infection.

^g^PrEP: pre-exposure prophylaxis.

^h^PrEP use in the lifetime was reported by 1 respondent with self-reported HIV-positive status.

### Unadjusted Associations of Online Partner Seeking

Some health-protective behaviors were more common among those using online tools (Table 2). The odds of reporting online partner seeking were lower for those who practiced condomless insertive (OR 0.26, 95% CI 0.10-0.70; *P*=.008) and receptive (OR 0.30, 95% CI 0.12-0.75; *P*=.01) anal intercourse with male partners. The odds of reporting online partner seeking were significantly higher among those who reported PrEP use in the lifetime (OR 3.73, 95% CI 1.27-10.96; *P*=.02).

Compared with nonusers, users of online tools for partner seeking reported a higher median number of recent male insertive anal sex (2 vs 1; OR 1.28, 95% CI 1.10-1.49; *P*=.001), receptive anal sex (2 vs 1; OR 1.19, 95% CI 1.05-1.33; *P*=.005), and oral sex partners (4 vs 1; OR 1.25, 95% CI 1.12-1.40; *P*<.001). In our sample, of the 252 respondents, 144 (57.1%) reported drug use in the past 6 months (Table 1); of these 144 respondents, 33 (22.9%) reported daily drug use. Online partner seeking was more likely to be observed among those who reported recent drug use (OR 2.04, 95% CI 1.16-3.57; *P*=.01) as well as substance use before or during sex (OR 2.34, 95% CI 1.34-4.09; *P*=.003). Of the 181 users of online tools, 65 (35.9%) reported recent substance use before or during sex with people they met through social media (Table 1). Although not considered for further analysis because of small cell sizes, engagement in group sex was significantly higher among users than among nonusers of online tools for partner seeking (OR 2.93, 95% CI 1.36-6.32; *P*=.006). Differences in reporting history of HIV (OR 0.87, 95% CI 0.29-2.58; *P*=.80) or STI (OR 1.51, 95% CI 0.79-2.87; *P*=.21) diagnoses were insignificant between users and nonusers.

**Table 2:** Unadjusted and adjusted comparisons of men who have sex with men who are users versus nonusers of online tools for partner seeking by demographic and behavioral characteristics (N=252).

Characteristics or behavior				Users^a^^,^^b^ (n=181)		Nonusers^a^ (n=71)		OR^c^ (95% CI)		*P* value
**Demographic characteristics**										
	Age (years), median (IQR)			25.2 (22.5-28.8)		27.1 (24.1-29.5)		0.94 (0.88-1.00)		.07
	Income in the past 30 days (US $), median (IQR)			1500 (750-2500)		1750 (1000-2500)		0.99 (0.98-1.00)^d^		.04
	**Gender, n (%)**									
		Man	176 (97.2)		68 (95.8)		Reference		N/A^e^	
		Other	5 (2.8)		3 (4.2)		0.64 (0.15-2.77)		.55	
	**Race and ethnicity, n (%)**									
		White and non-Hispanic	145 (82.4)		60 (87)		Reference		N/A	
		Other	31 (17.6)		9 (13)		1.43 (0.64-3.17)		.39	
	**Sexual orientation, n (%)**									
		Gay or mostly gay	152 (84)		63 (90)		Reference		N/A	
		Other (bisexual or heterosexual or pansexual or queer)	29 (16)		7 (10)		1.71 (0.72-4.12)		.23	
	**Education, n (%)**									
		≤GED^f^ or high school graduate	25 (13.8)		11 (15.5)		0.87 (0.41-1.89)		.73	
		≥Some college or college graduate	156 (86.2)		60 (84.5)		Reference		N/A	
	**County of residence^g^, n (%)**									
		Metropolitan	159 (87.9)		55 (77.5)		2.10 (1.03-4.29)		.04	
		Nonmetropolitan	22 (12.2)		16 (22.5)		Reference		N/A	
**Recent behaviors (past 6 months)**										
	**Type of anal sex with male partners, n (%)**									
		Only insertive (ie, participant was always in insertive position)	44 (24.4)		15 (21.1)		Reference		N/A	
		Only receptive (ie, participant was always in receptive position)	25 (13.9)		19 (26.8)		0.45 (0.19-1.04)		.06	
		Insertive or receptive	111 (61.7)		37 (52.1)		1.02 (0.51-2.05)		.95	
	Number of male insertive anal sex partners^h^, median (IQR)			2 (1-5)		1 (0-1)		1.28 (1.10-1.49)		.001
	Number of male receptive anal sex partners^i^, median (IQR)			2 (1-5)		1 (1-1)		1.19 (1.05-1.33)		.005
	Number of male oral sex partners, median (IQR)			4 (2-10)		1 (1-2)		1.25 (1.12-1.40)		<.001
	Condomless insertive anal intercourse^h^, n (%)			122 (75.3)		58 (92.1)		0.26 (0.10-0.70)		.008
	Condomless receptive anal intercourse^i^, n (%)			115 (73.3)		55 (90.2)		0.30 (0.12-0.75)		.01
	Engagement in group sex, n (%)			54 (29.8)		9 (12.7)		2.93 (1.36-6.32)		.006
**Recent substance use** **(past 6 months)** **, n (%)**										
	Used drugs to get high			113 (62.4)		31 (44.9)		2.04 (1.16-3.57)		.01
	Alcohol or any illicit drug use before or during sex			119 (65.8)		32 (45.1)		2.34 (1.34-4.09)		.003
**Lifetime behaviors, n (%)**										
	Ever tested positive for STIs^j^			56 (31.3)		16 (23.2)		1.51 (0.79-2.87)		.21
	Have been tested for HIV			158 (87.3)		57 (80.3)		1.69 (0.81-3.50)		.16
	Ever tested positive for HIV			12 (7.7)		5 (8.8)		0.87 (0.29-2.58)		.80
	Antiretroviral therapy uptake			12 (6.7)		5 (7)		0.95 (0.32-2.80)		.92
	PrEP^k^ use			33 (18.2)		4 (5.6)		3.73 (1.27-10.96)		.02

^a^Percentages may not add to total 100% because of rounding to 1 decimal. Sample sizes for some variables could vary because of missing data as well as refuse to answer or not applicable (n/a) responses.

^b^Includes all respondents who answered *yes* to the questions about using online tools to meet partners for sex, dating, or for both purposes (n=181).

^c^OR: odds ratio.

^d^Per US $100 increase.

^e^N/A: not applicable.

^f^GED: General Educational Development Test.

^g^US Department of Agriculture 2013 Rural-Urban Continuum Codes were used to classify counties of residence as metropolitan (5 counties, codes 1-3) and nonmetropolitan (6 counties, codes 4-6).

^h^With whom participants were in the insertive position.

^i^With whom participants were in the receptive position.

^j^STI: sexually transmitted infection.

^k^PrEP: pre-exposure prophylaxis.

### Adjusted Associations of Online Partner Seeking

Adjusting for demographics (Table 3), PrEP use in the lifetime as well as the number of recent male insertive or receptive anal sex partners and condomless insertive and receptive anal intercourse events were associated with lower odds of online partner seeking (aOR 0.22, 95% CI 0.07-0.68; *P*=.009 and aOR 0.25, 95% CI 0.10-0.66; *P*=.005, respectively). Adjusting for demographics, substance use before or during sex was associated with increased odds for online partner seeking (aOR 2.50, 95% CI 1.41-4.44; *P*=.002).

Our sensitivity analysis, while adjusting for residence in metropolitan and nonmetropolitan counties, provided similar results regarding significance and magnitude of associations, indicating lower odds of online partner seeking among those who had reported condomless insertive or receptive anal sex and higher odds of online partner seeking for those who reported substance use before or during sex (Multimedia Appendix 1). Sensitivity analyses also showed that there were no differences in online partner seeking between residents of metropolitan counties and those of nonmetropolitan counties.

**Table 3:** Adjusted comparisons of men who have sex with men who are users versus nonusers of online tools for partner seeking by sexual and drug-related behavioral characteristics.

Characteristic or behavior				Model 1 (n=214)				Model 2 (n=209)				Model 3 (n=245)		
				aOR^a^ (95% CI)		*P* value		aOR (95% CI)		*P* value		aOR (95% CI)		*P* value
**Demographic characteristics and** **PrEP^b^ use**														
	Age		0.89 (0.82-0.97)		.007		0.96 (0.89-1.04)		.34		0.94 (0.87-1.00)		.06	
	**Race and ethnicity**													
		White and non-Hispanic	Reference		N/A^c^		Reference		N/A		Reference		N/A	
		Other	0.77 (0.29-2.06)		.61		0.91 (0.35-2.38)		.85		1.40 (0.61-3.23)		.43	
	**Education**													
		≤GED^d^ or high school graduate	0.46 (0.16-1.35)		.16		1.10 (0.39-3.10)		.86		0.64 (0.27-1.56)		.33	
		≥Some college or college graduate	Reference		N/A		Reference		N/A		Reference		N/A	
	**Ever used** **PrEP** **in the lifetime**													
	Yes		4.26 (1.32-13.77)		.02		3.67 (1.00-13.51)		.05		N/A		N/A	
	No		Reference		N/A		Reference		N/A		N/A		N/A	
**Recent behaviors (past 6 months)**														
	Number of male insertive anal sex partners^e^		1.31 (1.11-1.55)		.001		N/A		N/A		N/A		N/A	
	Number of male receptive anal sex partners^f^		N/A		N/A		1.20 (1.05-1.39)		.008		N/A		N/A	
	Condomless insertive anal intercourse^e^		0.22 (0.07-0.68)		.009		N/A		N/A		N/A		N/A	
	Condomless receptive anal intercourse^f^		N/A		N/A		0.25 (0.10-0.66)		.005		N/A		N/A	
	Alcohol or any illicit drug use before or during sex		N/A		N/A		N/A		N/A		2.50 (1.41-4.44)		.002	

^a^aOR: adjusted odds ratio.

^b^PrEP: pre-exposure prophylaxis.

^c^N/A: not applicable.

^d^GED: General Educational Development Test.

^e^With whom participants were in the insertive position.

^f^With whom participants were in the receptive position.

## Discussion

### Principal Findings

This cross-sectional study of 252 young adult MSM residing in small and midsized towns in Central Kentucky revealed that 181 (71.8%) respondents had used online tools to meet partners for sex, dating, or for both purposes. Of these 181 respondents, 166 (91.7%) had used online tools to meet partners for sex (n=45, 27.1%, for sex only and n=121, 72.9%, for sex and dating) and 136 (75.1%) had used online tools to meet partners for dating (n=15, 11%, for dating only and n=121, 89%, for sex and dating). In our study, those who practiced online partner seeking reported a higher number of male insertive and receptive anal sex partners in the past 6 months and many reported substance use before or during sex. MSM who used online tools for partner seeking were more likely to report condom use during anal intercourse with male partners than their counterparts who did not practice online partner seeking.

The percentage of men using online tools for partner seeking in our study (181/252, 71.8%) is at the high end of what has been observed in other studies in large urban settings (64.6%-72.1%) [10,20,70]. These findings are consistent with previous research suggesting that because of the limited number of MSM-friendly venues for entertainment and social interaction [33,39], MSM residing outside of large metropolitan areas might use online tools for partner seeking even more to lower the risk of stigma [33,35,36,38] associated with their sexual identity. It is worth noting that our study sample consisted of a substantial proportion of MSM residing outside of major urban areas compared with samples from other studies on the topic that have almost exclusively focused on large urban settings [7,11,18,22,25-28]. Interestingly, despite higher proportions of users of online tools among MSM residing in metropolitan counties, our adjusted analysis revealed no differences in online partner seeking between residents of metropolitan areas and those of nonmetropolitan areas.

In line with previous research [20], including among rural MSM [71], online partner seeking for dating was less common than that for sex among users of online tools in our study: 24.9% (45/181) reported use of online tools exclusively for sex and only 8.3% (15/181) used online tools to seek partners specifically for dating (ie, not necessarily just for sex). Most of the users of online tools (121/181, 66.9%) were interested in seeking partners for both dating and sex. It is likely that MSM who reported online partner seeking for both purposes were predominantly interested in casual or one-time sex partners and, as seen in other studies, were also “dating for fun” [72]. Previous research shows that engagement in risk behaviors may vary by the intention of online partner seeking [73]. Bauermeister et al [73] showed that MSM who frequently practiced online partner seeking specifically for casual sex engaged more in condomless sex acts than MSM who frequently sought dating partners or MSM who rarely practiced online partner seeking. Some authors [14,18,26] have presumed that people who use online tools to look for sexual encounters, especially for casual sex partners, may have higher levels of sensation seeking [74] associated with lower levels of self-control [75]: the 2 characteristics that are generally related to sexual risk taking among MSM [66,75-80]. Because of the small numbers of MSM who reported use of online tools exclusively for dating or for sex, there was insufficient statistical power to detect differences in associations of risk behaviors with online partner seeking by the 2 intentions and future studies are recommended.

Consistent with previous research from large urban centers [10,11,22,67], online partner seeking among MSM in our study was associated with several risk behaviors, including an increased number of male insertive and receptive anal sex partners in the past 6 months before and after adjusting for PrEP use in the lifetime as well as substance use before or during sex. Similar to previous studies [81,82], unadjusted analyses revealed that a larger proportion of users of online tools reported recent drug use and engagement in group sex. Although online partner seeking was associated with various risk factors, our study also showed that some protective behaviors were more prevalent among users of online tools versus nonusers. This finding is important given that some HIV care providers have negative perceptions of MSM who report online partner seeking, and who are assumed to engage in increased sexual risk behaviors, without acknowledgment that it may also be associated with protective behaviors [83]. This study’s findings and those of others [9,67,81,82,84,85] demonstrating the association of online partner seeking not only with sexual risk but also with protective behaviors is important to help HIV care providers understand the nuances of online partner seeking and thereby have more open conversations about online partner seeking with their clients. Past studies show equivocal results about the relationship between online partner seeking and condomless sex, with some studies among MSM from rural areas or large cities showing higher odds [30,31,86,87], whereas others report lower odds [10-12] or no associations [85,88]. Our results revealed lower odds for online partner seeking among those who reported recent condomless insertive and receptive anal sex. In a study on MSM residing outside of large urban areas, Bowen et al [71] suggested that in high-risk situations, such as meeting relatively unknown partners online, MSM tend to frequently use condoms. However, with primary partners or men to whom they feel attracted, MSM may engage in condomless sex even if they are not very familiar with their partner and are unaware of their partner’s HIV status [71]. More research using relationship-level data will be valuable in better understanding how online partner seeking may play a role in affecting this association.

Evidence shows that MSM who seek partners online are more likely to report HIV [9] or STI [85] testing and willingness to use PrEP [84,89]. Consistent with previous research, we observed more (although not statistically significantly) HIV testing among users and nonusers of online tools in unadjusted analyses. MSM are recommended to be tested for HIV if they have had more than 1 sex partner since their last HIV test [90]. Therefore, the association between HIV testing and online partner seeking may be explained by increased sexual activity among users of online tools in our study and, likely, by more awareness of, and interest in, sexual health. The unadjusted analysis also revealed that a larger proportion of MSM who used online tools reported having ever taken PrEP than those who had not used online tools. However, the small number of participants who had received PrEP among nonusers of online tools prevented us from deriving consistent inferences in the adjusted analysis (ie, there were only marginally significant associations in the model for condomless receptive anal sex). In our sample, only 15.3% (36/235) of the MSM potentially eligible for PrEP use (after excluding 17 respondents who were aware of their HIV-positive status) had ever used PrEP, which is lower than the percentages for PrEP use reported from studies on MSM residing in large urban areas [91] but higher than the percentages reported in other studies on MSM from rural areas in the American South [89]. According to cross-sectionally collected data from 20 urban locations in the United States and territories as part of the National HIV Behavioral Surveillance conducted by the Centers for Disease Control and Prevention [92], there was a notable increase in PrEP awareness (from 60% to 90%) and use (from 6% to 35%) from 2014 to 2017 among MSM who are at risk for HIV infection [91]. Some authors have presumed that recent increases in PrEP awareness and use might be partly attributable to HIV prevention campaigns on social media [91]. Notably, an analysis of data from the American Men’s Internet Survey [93] revealed that the likelihood of PrEP awareness and use was lower in rural and nonmetropolitan regions than in urban areas [94]. These findings in combination with our study’s results indicate a need to increase PrEP awareness and uptake among nonurban MSM and highlight the need to explore social media as a strategy to do so.

### Limitations

First, this was a cross-sectional survey. Second, data are subject to recall and self-report biases, although the use of an online self-administered questionnaire should mitigate this limitation because it is typically better suited for collection of sensitive data [95,96], as should the federal certificate of confidentiality which was explained to participants in the consent and survey; this provides additional protection to the data. Third, although data on antiretroviral therapy uptake were collected, data on viral load were not, leaving us unable to describe the sample in terms of viral load suppression. Fourth, this was a convenience sample of young adult MSM residing in Central Kentucky and the sample may not be representative of all young adult MSM, especially those of lower educational attainment. Many resided in a midsized college town and were still in school or have already received their degree. A number of studies have shown that MSM who seek partners online are more likely to have higher level of education and be of higher income and, thus, are more likely to access the internet and mobile apps [9]. Therefore, our analyses were adjusted for confounding by educational attainment.

In addition, the study recruited participants through multiple sources, including but not limited to social media, leading to potential overrepresentation of men who frequently use online tools for partner seeking. A study on nonurban MSM by Bowen et al [71] and a meta-analysis by Liau et al [13] showed that more men recruited online practiced internet partner seeking than men recruited offline. Of the 252 participants in our study, 64 (25.4%) were recruited at a pride festival. Some suggest that men who attend pride festivals are more open about their sexuality and engagement in same-sex sexual activity [67]. Bowen et al [71] reported that more nonurban MSM recruited through conventional sampling methods were in long-term monogamous relationships than men recruited solely on the internet [71]. However, it is worth noting that only 20.6% (52/252) reported social media as a way in which they had learned about the survey. Some (79/252, 31.3%) of the participants reported learning about the study through more than 1 method (eg, through peer referral and seeing it on social media); hence, responses regarding the recruitment methods were not mutually exclusive. Furthermore, the proportion of MSM practicing online partner seeking did not differ significantly by the type of recruitment method, and thus we did not adjust for recruitment type in the analysis. Finally, the study did not distinguish between different types of social networking and dating websites or smartphone-based geosocial networking apps that were reported by respondents but rather focused on the overall use of various online tools for partner seeking.

Despite these limitations, our study is among the first to describe the experiences of MSM residing outside of major cities, a population that is underrepresented in research on the topic. We believe that the *lack* of differences between our findings and those of national and urban studies are as informative as the differences themselves because they provide insights into the extent to which MSM residing in small and midsized towns are similar to those residing in large cities and, therefore, may respond similarly to similar intervention approaches or experience similar challenges.

### Conclusions

To our knowledge, this study is one of the first of its kind to examine online partner seeking and associated sexual behaviors among MSM from small and midsized towns and the first one in Kentucky: 71.8% (181/252) had used online tools to meet partners for sex, dating, or for both purposes. Consistent with research on MSM from larger metropolises, online partner seeking was associated with some sexual risk behaviors such as increased number of anal sex partners and substance use before or during sex. These results provide insights regarding the content of targeted HIV risk-reduction internet- or mobile-based interventions among MSM who practice online partner seeking. However, unlike most studies among MSM from rural areas or large urban centers, we observed positive associations of online partner seeking with some protective behaviors such as condom use during insertive and receptive anal intercourse. This suggests that more tailored interventions are needed to reduce the risk of HIV transmission associated with condomless anal intercourse among MSM who do not practice online partner seeking.

## Appendix

Multimedia Appendix 1 Adjusted comparisons of users versus nonusers of online tools on sexual and drug-related behavioral characteristics with and without adjusting for metropolitan residence.

## Abbreviations

aOR: adjusted odds ratio
GED: General Educational Development Test
MSM: men who have sex with men
OR: odds ratio
PrEP: pre-exposure prophylaxis
PWID: persons who inject drugs
STI: sexually transmitted infection
