# Supplementary material for: Online Partner Seeking and Sexual Behaviors Among Men Who Have Sex With Men From Small and Midsized Towns: Cross-sectional Study
Source: JMIR Form Res. 2022 Jun 10;6(6):e35056. doi: 10.2196/35056 (PMC9233248; doi:10.2196/35056)
Supplement: Multimedia Appendix 1 [file formative_v6i6e35056_app1.docx]

Table S1. Adjusted comparisons of users versus nonusers of online tools by sexual and drug-related behavioral characteristics *with* and *without* adjusting for metropolitan residence

| Characteristic or behavior | aOR^a^ (95% CI), *P value*  ***WITHOUT*** *Metro indicator variable* | | | aOR (95% CI), *P value*  ***WITH*** *Metro indicator variable* | | |
| --- | --- | --- | --- | --- | --- | --- |
|  | Model 1 (n=214) | Model 2 (n=209) | Model 3 (n=245) | Model 1 (n=214) | Model 2 (n=209) | Model 3 (n=245) |
|  |  |  |  |  |  |  |
| **Demographic characteristics and PrEP use** |  |  |  |  |  |  |
| Age | 0.89 (0.82-0.97),  .007 | 0.96 (0.89-1.04),  .34 | 0.94 (0.87-1.00),  .06 | 0.885 (0.81-0.96),  .005 | 0.96 (0.89-1.04), .30 | 0.93 (0.87-1.00),  .048 |
| Race/ethnicity |  |  |  |  |  |  |
| White, non-Hispanic | Reference | Reference | Reference | Reference | Reference | Reference |
| Other | 0.77 (0.29-2.06),  .61 | 0.91(0.35-2.38),  .85 | 1.40 (0.61-3.23),  .43 | 0.76 (0.285-2.01),  .58 | 0.89(0.34-2.31),  .80 | 1.35 (0.58-3.11), .49 |
| Education |  |  |  |  |  |  |
| ≤GED^b^/High school graduate | 0.46 (0.16-1.35),  .16 | 1.10 (0.39-3.10),  .86 | 0.64 (0.27-1.56),  .33 | 0.44 (0.15-1.308),  .14 | 1.03 (0.37-2.93), .94 | 0.61 (0.25-1.49),  .28 |
| ≥Some college/college graduate | Reference | Reference | Reference | Reference | Reference | Reference |
| Ever used PrEP^c^ in the lifetime |  |  |  |  |  |  |
| Yes | 4.26 (1.32-13.77)  .02 | 3.67 (1.00-13.51)  .05 |  | 4.18 (1.29-13.486),  .02 | 3.63 (0.98-13.38),  .05 |  |
| No | Reference | Reference |  |  |  |  |
| **Reside in metropolitan area** |  |  |  |  |  |  |
| Yes |  |  |  | 1.54 (0.63-3.77),  .34 | 1.71 (0.71-4.13),  .24 | 1.76 (0.83-3.77),  .14 |
| No |  |  |  | Reference | Reference | Reference |
| **Recent behaviors (past 6 months)** |  |  |  |  |  |  |
| Number of male insertive anal sex partners^d^ | 1.31 (1.11-1.55)  .001 |  |  | 1.30 (1.11-1.54),  .002 |  |  |
| Number of male receptive anal sex partners^e^ |  | 1.20 (1.05-1.39)  .008 |  |  | 1.19 (1.04-1.36),  .01 |  |
| Condomless insertive anal intercourse^d^ | 0.22 (0.07-0.68)  .009 |  |  | 0.22 (0.07-0.70),  .01 |  |  |
| Condomless receptive anal intercourse^e^ |  | 0.25 (0.10-0.66)  .005 |  |  | 0.25 (0.09-0.65),  .005 |  |
| Alcohol or any illicit drug use before or during sex |  |  | 2.50 (1.41-4.44)  .002 |  |  | 2.37 (1.32-4.23), .004 |

^a^aOR: adjusted odds ratio.

^b^PrEP: pre-exposure prophylaxis.

^c^GED: General Educational Development Test.

^d^With whom participants were in the insertive position.

^e^With whom participants were in the receptive position.
